# Improving the Efficiency of Electrical Discharge Machining of Special-Purpose Products with Composite Electrode Tools

**DOI:** 10.3390/ma14206105

**Published:** 2021-10-15

**Authors:** Timur Rizovich Ablyaz, Evgeny Sergeevich Shlykov, Karim Ravilevich Muratov

**Affiliations:** Mechanical Engineering Faculty, Perm National Research Polytechnic University, 614000 Perm, Russia; kruspert@mail.ru (E.S.S.); karimur_80@mail.ru (K.R.M.)

**Keywords:** electrical-discharge machining, Ti-6Al-4V, metal matrix composite, surface characterization, wear resistance, corrosion resistance

## Abstract

The article is devoted to increasing the efficiency of electrical discharge machining of special-purpose items with composite electrode tools. The subject of research is the parameter of the roughness of the processed surface and the work of the electro-discharge machining (EDM) of 40Crsteel in various modes of electrical discharge machining. The aim of the work is to increase the efficiency of the process of copy-piercing electrical discharge machining of parts introduced into the composition of a special-purpose product and the use of electrode tools with the introduction of 20% graphite. Experimental studies were carried out using the method of a full factorial experiment with a subsequent regression analysis. The experiments were carried out using a copy-piercing Smart CNC EDM machine, a tool electrode, and a profile composite electrode. Empirical dependencies were established, reflecting the relationship between the processing modes, productivity, and surface roughness parameter after processing. A theoretical model for calculating the roughness parameter was developed, which makes it possible to predict the quality of the processed surface with a reliability of 10–15%. To ensure the required ratios of the quality of the processed surface at the maximum performance indicators, technological recommendations were obtained, as a result of which a 35% reduction in machine time was achieved when processing the “screw” part with the required indicators of surface quality.

## 1. Introduction

When creating products operating under conditions of increased loads, alloy steels with increased physical and mechanical properties are used. The technological requirements for ensuring the accuracy of the manufacture of such products are increasing. The development of three-dimensional modeling technologies makes it possible to design products with complex spatial shapes in order to increase their operational properties.

The creation of new, technically more advanced products forces designers to use progressive materials, tighten requirements for geometry accuracy, and use spatially complex structures in a design [1,2,3]. For the manufacture of spatially complex parts by traditional methods of machining, as a rule, a volumetric preparation of production is required, which makes the process of manufacturing a product long and costly. In addition, in some cases, blade processing cannot be implemented due to the impossibility of providing a kinematic processing scheme due to the complexity of the geometry of the workpiece [4].

In this regard, more universal methods of physical and chemical processing are promising, allowing for the manufacture of parts with complex geometry by the method of three-dimensional copying. One of the widely used such methods is the technology of copy-piercing electro-discharge machining (EDM), the advantage of which, in comparison with mechanical processing, is the ability to perform complex geometry with high accuracy and a high frequency of surface roughness, as well as the ability to process materials regardless of their hardness [5,6,7,8].

In the manufacturing of parts that make up special-purpose products (ISN), the EDM technology is widely used in the processing of blind curved grooves, deep holes of a small diameter, and internal splines and teeth. Analysis of the materials of ISN parts processed by the CPEE method showed that alloyed chromium-containing steels are mainly used for their manufacture. The presence of chromium increases the electrical discharge resistance of the material, which leads to a decrease in the productivity of the EDM process and intensive wear of the tool electrode (ET) [9,10].

Analysis of the literature has shown that a number of composite materials have been developed to improve the operational properties of ETs. The most promising such material is a composite material of a pseudo-alloy system of copper–colloidal graphite (CCG) with a graphite content of 20%; however, ETs from this material are not widely used in the EDM of special parts. This is due to the fact that the operational properties of ETs from a given composite material in the case of EDM for chromium-containing steels have been little studied; hence, there are no recommendations on the appointment of processing modes [11,12,13].

Relevant scientific and technical problems are the study of the operational properties of ETs from CCG with a graphite content of 20% at the EDM of chromium-containing steels used to create special parts, obtaining empirical models that make it possible to predict the performance of processing and the quality of the processed surface, and studying the effect of the ET material on the structure and properties of the processed surfaces of chrome-containing steels.

The aim of this work is to increase the efficiency of the process of copy-piercing electrical discharge machining of parts included in the composition of a special-purpose product by using electrode tools made of a composite material such as a pseudo-alloy system of CCG with a graphite content of 20%.

### Related Work

At present, domestic and foreign scientists have developed a number of wear-resistant composite materials for electrical purposes, but the study of the structure and properties in order to increase the erosion resistance of the electrode tool material has not been systematic and is not sufficient. Promising refractory additives such as carbon nanotubes and titanium carbo-silicide for the manufacture of instrument electrodes have not been fully considered. Leading universities, including the Technical University of Lisbon (Portugal), the Polytechnic Institute of LETria (Portugal), Texas A&M University (United States), the Central Research Engineering University CSIR-CMERI (India), Tula State University (Russia), and the Industrial University of Tokyo IIS (Japan), are engaged in the development of technology for the manufacture of electrode tools using powder metallurgy. The problem of creating electrode tools with increased electro-erosive properties has been discussed over the past 7 years at conferences and symposia devoted to electrophysical processing methods, such as the Conference on Electro Physical and Chemical Machining (ISEM). In the works of the authors Pisarciuc [14] and Lin Gu et al. [15], methods for creating electrode tools using additive technologies are considered. The issues of modeling the quality indicators of processed surfaces, which allow us to optimize the processing parameters, have not been fully studied. The analysis carried out shows that the use of existing techniques for creating ETs does not allow for the creation of electrodes with high wear resistance of the complex-profile working surfaces of the electrode. Existing developments do not address the issue of processing high-alloy materials with increased heat resistance and high-temperature wear resistance. There are no scientifically substantiated data on the prediction of the processing process and the nature of the wear of composite ETs for EDM of chromium-containing steels. Higher energy concentrations in the work area lead to increased wear rates. In the field of developing electrodes for electro-erosive piercing of new compositions, judging by the publications, competitors include the Institute for Problems of Materials Science, named after the V.I. I.N. Frantsevich National Academy of Sciences of Ukraine (Ukraine), which is developing composite materials based on copper with the addition of tungsten carbide, boron, etc. The analysis performed suggests that, at present, there are no generally accepted models of the behavior of alloy steels subjected to pulsed electrophysical action with composite ETs. The data on the assessment of quality parameters when processing alloy steels with composite ETs are not fully reflected. We need to accumulate experimental data on the features of the formation of roughness on the surface of alloy steels after exposure to current pulses when using ETs from composite materials.

## 2. Materials and Methods

### 2.1. Materials

**Figure 1:**
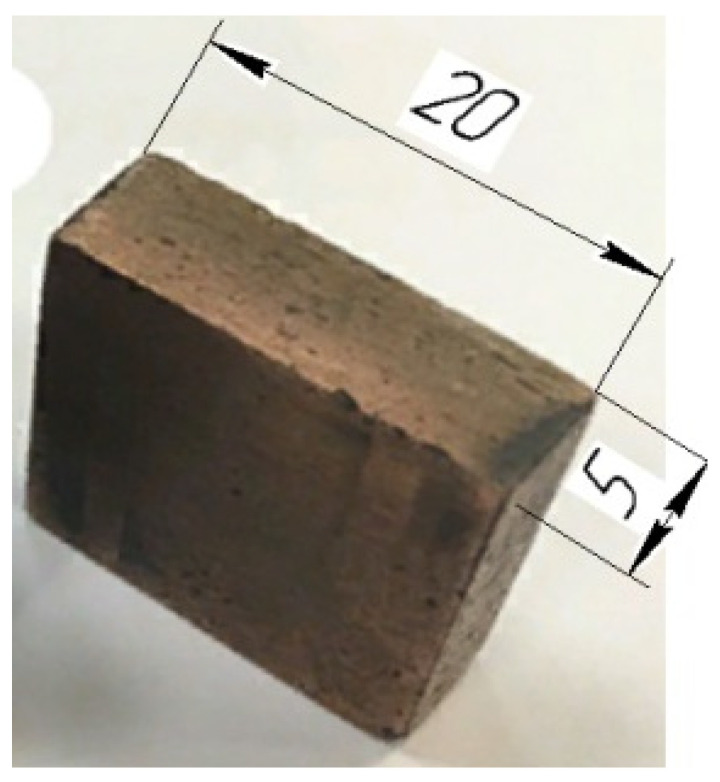
Composite material ET, mm.

To conduct the experiment, an ET with dimensions of 20 × 20 × 5 mm was made from a composite material based on copper powders and a preparation of dry colloidal graphite (Figure 1).

For the manufacture of an ET blank from a composite material, the method of powder metallurgy was used. PMS-1 copper powder was mixed with a preparation of S-1 dry colloidal graphite, in a ratio of 80/20, respectively, in a mixer for 4 h. Then, from the resulting composition, the workpiece was pressed on a P-125 press under a pressure of 600 MPa. After the first pressing, the billet was annealed in a vacuum furnace at a temperature of 700 °C, and repeated pressing and baking were performed at a temperature of 1070 °C. After receiving the workpiece, it was processed to the required dimensions by milling. The residual porosity was no more than 10%. A set of 40Cr low-alloy steel specimens were made to study the surface properties of chromium-containing steels as a function of ET material and to conduct a complete factorial experiment. The samples were heat-treated to a hardness of 400–650 HV.

### 2.2. Temperature Simulation

A simulation of a single discharge pulse from the surface of a composite material ET to the surface of a workpiece made of chromium–nickel steel was carried out. A sample of structural alloy steel, which is a sample in the form of a size10 × 20 × 40 mm plate, was placed in a dielectric liquid (transformer oil). The ET has a cylindrical shape of size 10 × 10 mm.

**Figure 2:**
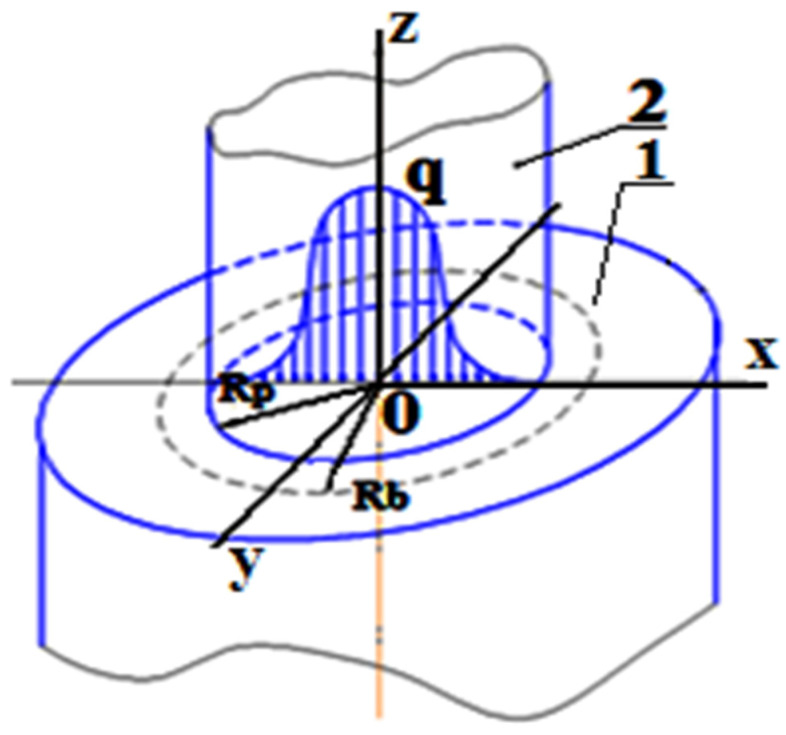
Plasma channel diagram: 1, steel ED; 2, plasma channel; q, heat flux (Watts); R_p_, radius of the heat source (m); R_b_, radius of the gas bubble (m).

As a result of the electrical breakdown of the dielectric, a plasma channel is created, which acts on the surface of the workpiece [16,17,18,19,20]. Modeling was carried out using the physical block “heating of solids”; therefore, a flat heat source is formed in the zone of influence of the plasma channel, which is designated by the radius R_p_ in Figure 2. At the initial moment of time, the plasma has neither mass nor a radius. There is a radiation of energy, which goes into the ET, the part, and the dielectric. The radius of the plasma channel over the course of time increases due to the high pressure inside the discharge channel in proportion to the radius of the zone of influence of the discharge channel [21].

Since the plasma channel has the shape of a cylinder, it is expedient to carry out modeling for a two-dimensional axisymmetric model. The mass of the plasma increases as its radiation evaporates and ionizes a small layer of liquid.

Over the course of time, the radius of the channel increases due to the high pressure inside the channel and the transformation of the dielectric into plasma. This transformation takes about 70% of the total energy [19,20].

As the plasma channel expands, its internal pressure decreases, while the pressure in the adjacent dielectric increases. Due to the short pulse duration, the effect of such a process is insignificant and can be ignored [22,23,24].

The continuity equation has the form:

(1) $$\left(\frac{\delta \rho_{0}}{\delta t}\right)+\frac{1}{r}\cdot \frac{\delta }{\delta r}\left(\rho_{0}\cdot r\cdot \nu_{r}\right)$$

where *t* is time (μs) and $\rho_{0}$ is the dielectric de(m/s). Thus, the expansion rate of the plasma channel can be described by the equation:

(2) $$\nu_{r}=\left(\frac{R_{p}}{r}\right)\cdot \left(\frac{dR_{p}}{dt}\right)$$

where $\;R_{p}$ is the radius of the plasma channel (m). If we consider the unsteady state of the plasma in the form of a thermodynamic system, then the energy balance has the form:

(3) $$I\cdot U\cdot F_{p}=\left(25I\right)\cdot \left(0.74\right)=\left(H-H_{0}\right)\cdot \left(\frac{dm}{dt}\right)+m\cdot \left(\frac{dH}{dt}\right)$$

where *I* is the current strength (A), *U* is the voltage (V), *F_p_* is the part of the power attributable to the plasma (Watts), *H* is the average plasma enthalpy, *m* is the plasma’s mass (kg), and *H*_0_ is the enthalpy surrounding the insulator.

Due to the short duration of the pulse (μs), the heat transfer in the liquid of the breakdown channel by convection and thermal conductivity is insignificant.

Taking into account the energy balance equation for the unsteady state of the plasma and the expansion rate of the plasma channel, when creating a theoretical model, one should set the initial parameters as follows: the radius of the plasma channel, 0.2 mm; energy, 106 Watts.

**Figure 3:**
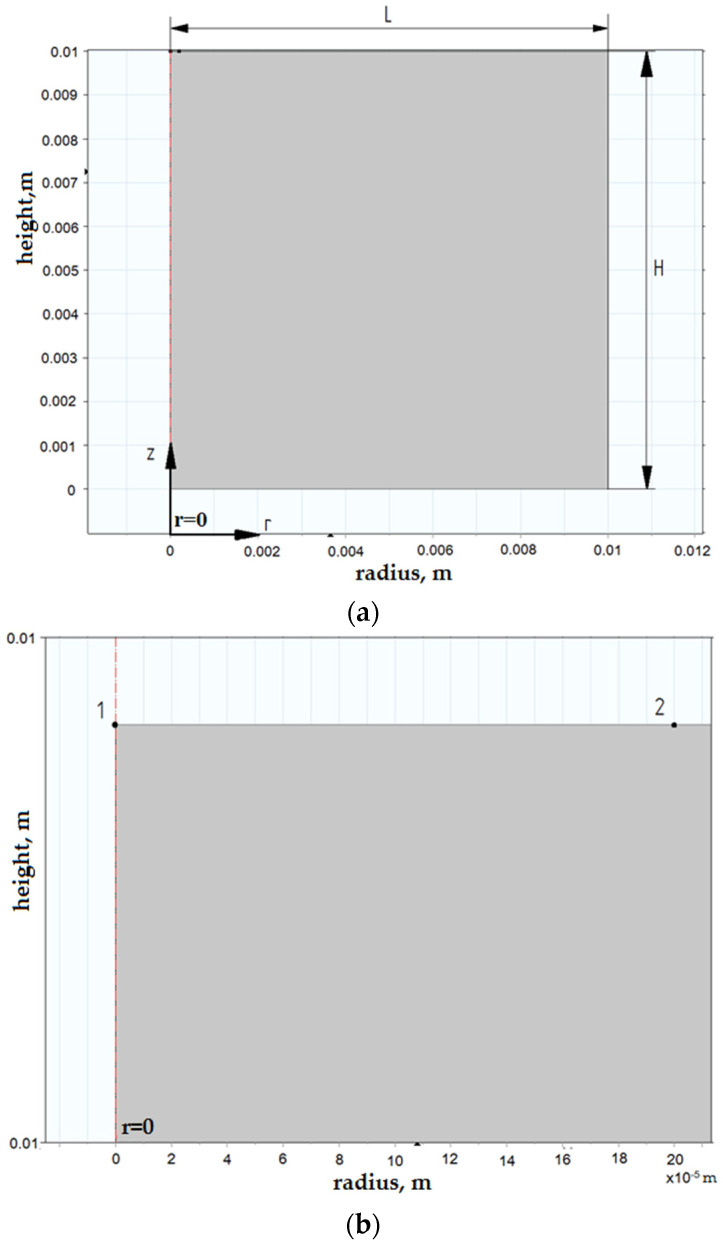
Geometric model of the part: (**a**) setting the geometry: H, cathode height (m); L, cathode radius (m); (**b**) points of the plasma channel: 1, the center of the plasma channel; segment 1–2, plasma radius.

To set the geometry, it is necessary to construct a rectangular shape imitating the cross-section of the electrode part (Figure 3a). We position the axis of symmetry of the model so that it passes through the center of the discharge channel. Figure 3b shows the points in the discharge zone.

To construct a geometric model of a single discharge, we assumed that the values were L = 10 mm, H = 10 mm, and segment 1–2 = 0.2 mm. Modeling was performed in the mode of axial symmetry.

Materials for the models were selected using the Comsol Multiphysics library. Structural 40Cr alloy steel from the group of chromium-containing steels was selected as the material of the workpiece. The thermophysical parameters of 40Cr steel are given in Table 1.

Before the electrical discharge, the initial temperature of the ET and DE was set to:

T |_t__=0_ = T_0_ (4)

where T_0_ = 293 K is the ambient temperature.

**Figure 4:**
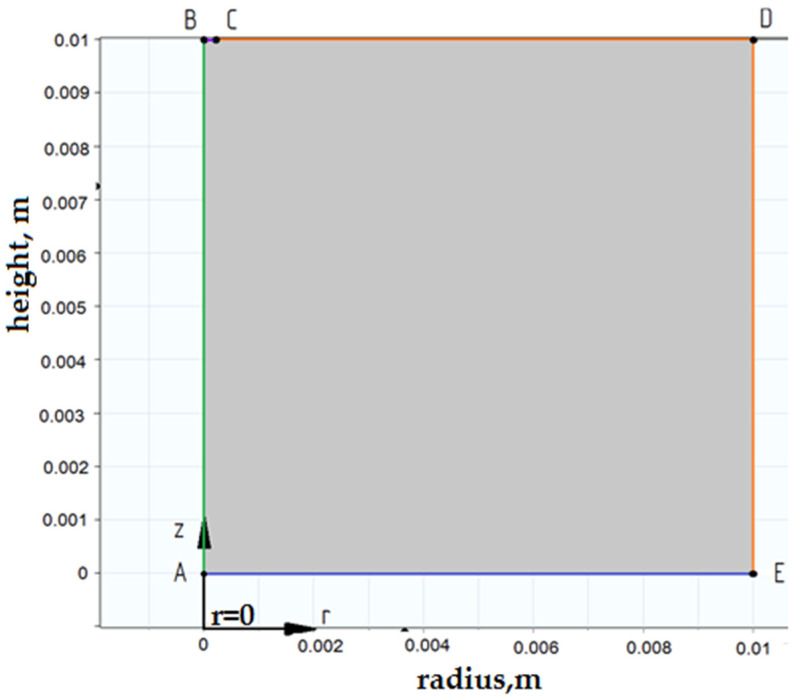
Setting the boundary conditions.

Boundary conditions in Comsol Multiphysics were selected using the Boundary Selection tool (Figure 4). We set the condition of axial symmetry along the boundary AB (r = 0). RG washes the boundaries of the parts CD and DE. The properties of the RF, which fills the interelectrode gap, affect the formation and development of the discharge channel.

The value of the breakdown gap at the selected amplitude of motion was determined by the properties of the working fluid. With an increase in the dielectric properties of the medium, the size of the gap decreases, the effect of the polarity is greater, and the removal of erosion products from the treatment zone is much worse. Equation (1), taking into account thermal constancy, has the form:

(5) $$n\cdot \left(q_{1}-q_{2}\right)=0;\;q_{i}=-k_{i}𝛻T_{i},$$

where *q* is the heat flux, $k_{i}$ is the heat transfer coefficient, $𝛻T_{i}$ are the temperatures produced at a point, and *n* is the normal vector. The breakdown channel energy is absorbed by the metal electrodes and the surrounding dielectric. Let us set the condition of thermal insulation at the border AE, through which heat diffusion into the environment is impossible:

(6) −n*(−k𝛻T)=0

The zone of influence of the plasma channel is determined by the boundary BC at the place where the heat flux *q* acts. The distribution of the heat flux *q* (see Figure 2) flows according to the law of normal distribution (the Gaussian law).

The main characteristics of working pulses in EDM are such parameters as energy, average power, duty cycle, and pulse duration. We represent the pulse energy in an empirical form:

(7) $$W=P\cdot q\cdot t_{on}$$

where *P* is the average pulse power (*W*); *q* is the duty cycle; and $\;T_{on}$ is the pulse duration (μs). The average power is determined by the following formula:

(8) P=U·I

where *U* is the average voltage value (V); and *I* is the average value of the current strength (A). The duty cycle is the ratio of the pulse repetition period to the duration of one pulse:

(9) $$q=\frac{T}{T_{on}}$$

where *T* is the pulse repetition period. The pulse duration *T_on_* at a constant frequency *f* is equal to:

(10) $$T_{on}=\frac{1}{f\cdot q}$$

**Figure 5:**
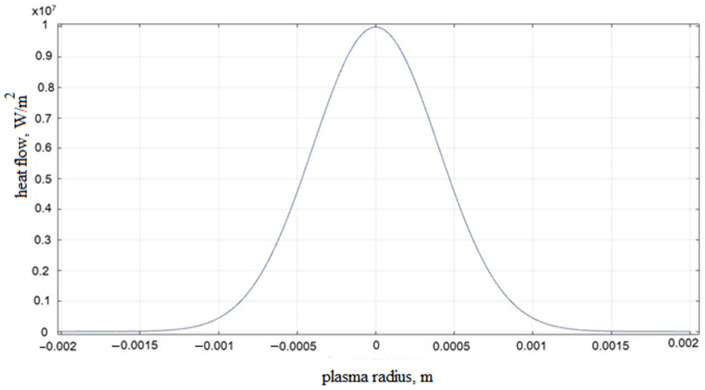
Distribution of the heat flux q depending on the radius of the plasma at the impulse action time of 0.0002 s.

We establish the dependence of all technological parameters of the KEEEO on the selected modes through the pulse energy, since it is one of the main operating parameters of the EDM instrument. In this model, thermophysical parameters are determined by the coordinates and temperature. To simulate the propagation of temperature fields in the investigated ET and workpiece, it is necessary to specify a flat heat source (see Figure 2). A flat heat source depends on the heat flux density *q*, which specifies the amount of heat that passes through the source area. The relationship between the magnitude of the heat flux and the parameters of the pulse signal is described by the following formula:

(11) $$q\left(t\right)=\eta \cdot \frac{4\cdot \left(I\left(t\right)\cdot U\left(t\right)\right)}{\pi \cdot R_{s}^{2}}$$

where η is the part of the discharge energy that enters the sample; and *R_s_* is the radius of the discharge channel (m). The heat flow function, which must be set, obeys the law of normal distribution. In Comsol Multiphysics, under the “Definitions” tab, we created a Gaussian pulse function (Figure 5).

The Gaussian distribution is used when evaluating products that are affected by a number of random factors, each of which has little impact on the resulting effect.

Let us define an equation that describes the nature of the distribution of the heat flux along the *x* coordinate:

(12) $$q\left(x\right)=\frac{1}{\sigma \left(t\right)\cdot \sqrt{2\pi }}\cdot e^{\frac{-{\left(x-mx\right)}^{2}}{2\sigma^{2}\left(t\right)}},$$

where *σ* is the standard deviation; *x* is a random variable; and *mx* is the mathematical expectation. The final form of the heat flow equation is:

(13) $$q\left(x,t\right)=\eta \cdot \frac{4\cdot \left(I\left(t\right)\cdot U\left(t\right)\right)}{\pi \cdot R_{s}^{2}}\cdot \frac{1}{\sigma \left(t\right)\cdot \sqrt{2\pi }}\cdot exp^{\frac{-{\left(x-mx\right)}^{2}}{2\sigma^{2}\left(t\right)}}$$

The heat flow function must be set in Comsol Multiphysics under the “Definitions” tab (Figure 5).

**Figure 6:**
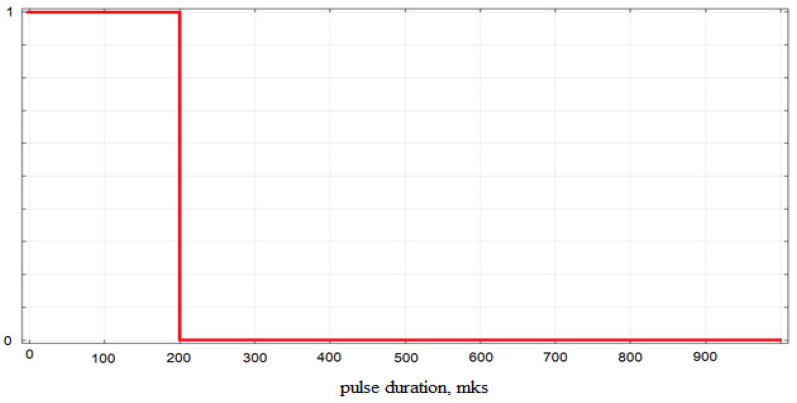
The pulse action function $T_{on}$.

To take into account the time of the impulse action ($T_{on}$) in the discharge channel according to the developed modeling technique, the impulse action function (“Step”) is introduced, which simulates the complex and variable nature of the EDM process and sets the pulse transit time $T_{on}$ = 200 μs (Figure 6).

Using the coefficient of the total heat flux (the “general inward heat flux”), we set a flat heating source in the Flux tab (cathode):

(14) $$q_{0}=heatflux\left(r\right)\cdot T_{on}\left(t\right)$$

where *heatflux*(*r*) is the function of the Gaussian distribution of the amount of heat flux at time (*t*) a long the coordinate (*r*), (W/m^2^); and $T_{on}$(*t*) is the function of the time step of the impulse action (μs).

Since the sample is washed by the working fluid (transformer oil), we will use the “Convective Heat Flux” simulation mode, which takes into account the convective heat flux:

(15) $$h\cdot T_{\mathrm{ext}}-T$$

where h is the coefficient of thermal conductivity (W/(m K));T_ext_ is the ambient temperature (°C);and T the body temperature (°C).

**Figure 7:**
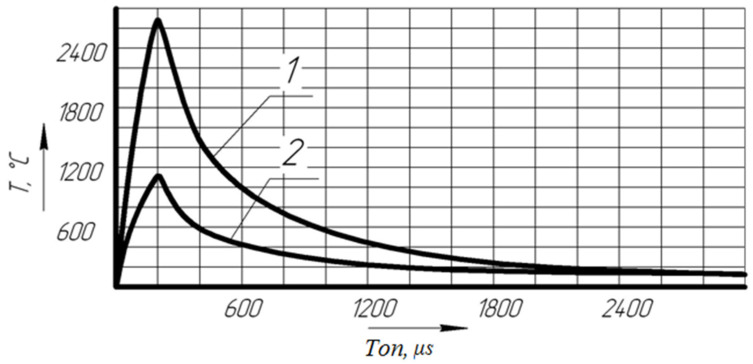
Graph of the change in temperature of the part when exposed to a single impulse. 1, temperature on the surface of the part in the center of the discharge; 2, temperature on the surface of the boundary of the radius of action of the plasma. T, temperature (°C); $T_{on}$, action time (μs).

As a result of the calculation, graphs of the temperature dependence in the zone of action of a single pulse in the center of the discharge and at the boundary of the radius of action of plasma on the cathode were obtained (Figure 7).

Analyzing the graph, we come to the conclusion that when exposed to a single discharge, the metal melts and some of it evaporates, leaving craters, while the temperature on the workpiece reaches its highest value at t = 200 μs. The process of changing the boundaries of melting and evaporation of a metal can continue for a certain time after the end of the heat flow due to the energy of the liquid phase, which has temperatures above the melting point, and the temperature of the upper layers exceeds the boiling point.

**Figure 8:**
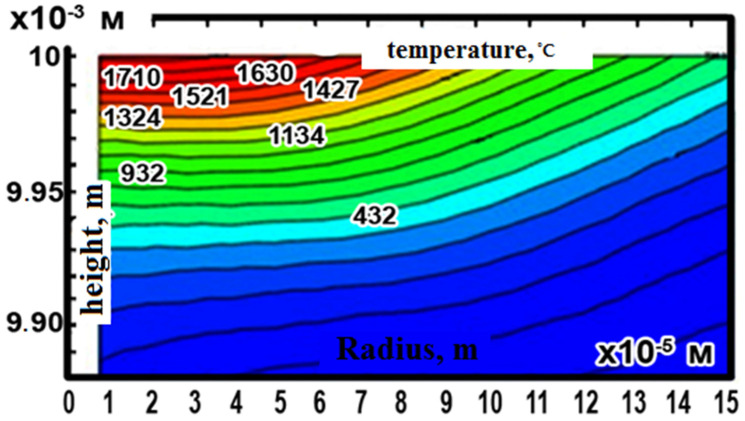
EM temperature fields after exposure to a pulsed discharge at time t = 200 μs.

As can be seen from Figure 8 which shows changes in temperature fields when exposed to a single pulse, the crater geometry generated in the part using a time-dependent heat source has the shape of a crater-bowl or dimple, that is, the bowl width is greater than the depth. At time t = 200 μs, the temperature effect in the hole reaches a maximum, then decreases. Under the influence of electrical impulses of a high energy concentration in the discharge zone, the metal on the electrode surface melts and evaporates, forming dimples close to a spherical shape.

### 2.3. Experimental Work

To study the influence of the modes of electrical discharge machining and the properties of the composite ET material on the roughness parameter of the processed surface of a workpiece made of 40Cr steel, a full factorial experiment was carried out.

To carry out a full factorial experiment, 15 experiments were performed. The experiments were carried out in accordance with the compiled planning matrix, depending on the selected processing modes.

When planning a factorial experiment by linear transformation of the coordinates of the factor space, the parameters were coded by linear transformation. The choice of scales along the axes was made in accordance with the units of the intervals of the selected variation factors, the docking of the zero point, and the origin of coordinates.

Since the main parameter influencing the formation of the cleanliness of the roughness of the treated surface during EDM is the pulse energy, which is determined by the processing modes, the following parameters were taken as variable factors: I, current strength (A); $T_{on}$, pulse duration (μs); and U, voltage (V).

The range of variation in the parameters was determined by the technological tables of the machine. The intervals for varying the parameters are presented in Table 2.

The number of necessary experiments depends on the number of levels of factors according to the following law:

(16) $$N=2^{k}+2k+1=15$$

where *N* is the number of experiments; and *k* is the number of factors.

To ensure the minimum influence of random factors on the EDM process, three parallel experiments were carried out. The initial parameters for constructing the planning matrix are presented in Table 3.

The planning matrix was compiled taking into account the coded factors with the value of the upper “stellar” shoulder α = 1.215 [25]. The dummy variable values were calculated using the formula:

(17) $${X^{\prime }}_{p}=X_{p}^{2}-\frac{2^{n}+2\cdot \alpha^{2}}{N}$$

where *p* is the factor number.

Based on the results of the experiments carried out for each row of the matrix, the average value of the optimization parameter was found:

(18) $${\bar{y}}_{j}=\frac{\sum_{i=1}^{k}y_{ji}}{h},$$

Where ${\bar{y}}_{j}$ is the value of the response function; *i* is the number of parallel experiments; *j* is the number of the experiment; and *h* is the number of parallel experiments.

For all points of the matrix, we calculated the sample variance to estimate the deviation from the average value of the optimization parameter:

(19) $$S_{j}^{2}=\frac{1}{h-1}\overset{k}{\underset{i=1}{\sum }}{\left(y_{ji}-{\bar{y}}_{j}\right)}^{2}$$

We calculated the error of the experiment:

(20) $$\sqrt{S_{j}^{2}}=S_{j}$$

In accordance with the method of factorial planning, the homogeneity of the variance was checked further.

## 3. Results

### 3.1. Factor Planning Results

The results of the factorial experiment are presented in Table 4.

We calculated the variance of reproducibility:

(21) $$S_{y}^{2}=\frac{\sum_{i=1}^{N}S_{j}^{2}}{N}=9.5803.$$

We calculated the Student’s coefficients and the variance of reproducibility. The obtained values are presented in Table 5.

At α = 0.05, the limit value of the Student’s criterion is t = 2.04. The insignificant coefficients b2, b3, b12, b13, b23, and b33 were discarded, and we obtained a refined model:

(22) $$y=14.72+3.84\cdot X_{1}-3.37\cdot X_{1}^{2}-1.38\cdot X_{2}^{2}$$

After calculating the coefficients and checking the significance, we determined the variance of the adequacy:

(23) $$S_{adequacy}^{2}=\frac{k\sum_{i=1}^{N}{\left({\bar{y}}_{j}-\hat{y_{j}}\right)}^{2}}{f_{adequacy}}=11.7032,$$

(24) $$f_{adequacy}=N-1=11,$$

(25) $$F_{\mathrm{estimated}}=1.2216<F_{tabl}=2.09.$$

Based on Formula (25), we can conclude that the model is adequate. We carried out the reverse replacement of the parameters for the transition to the experimental model according to the experimental plan and we obtained the final model, which has the following form

(26) $$Ra=5.02\cdot I-0.37\cdot I^{2}-0.0004\cdot T_{on}^{2}+0.076\cdot T_{on}-4.87$$

The final model of the dependence of the roughness parameter on the EDM modes, taking into account the pulse energy loss coefficient k, has the form:

(27) $$Ra=k\cdot \left(5.02\cdot I-0.37\cdot I^{2}-0.0004\cdot T_{on}^{2}+0.076\cdot T_{on}-4.87\right)$$

The resulting empirical model is a function of the response of two variables: current *I* (A) and pulse turn-on time $T_{on}$(μs). Accordingly, the cleanliness of the surface roughness of 40Cr steel, with the EDM ET made of a copper–colloidal graphite composite material, is influenced by a change in each of these parameters, as well as their combination, and is not affected by a change in the voltage parameter *U* (V).

**Figure 9:**
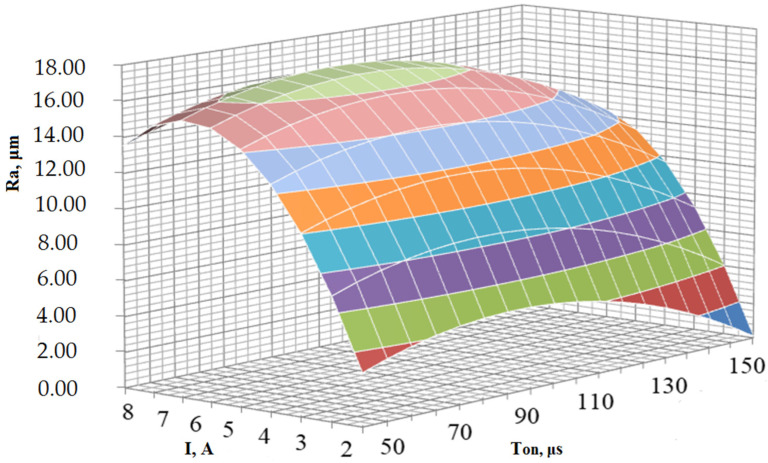
Influence of I (A) and $T_{on}$ (μs) on the cleanliness of the roughness of 40Crsteel subjected to EDM with a composite material ET.

To analyze the effect of EDM modes on the cleanliness of the roughness of 40Cr chromium-containing steel when processing the ET from a copper–colloidal graphite composite material, it is necessary to present an empirical model in the form of a hypersurface (Figure 9).

From the graph of the hypersurface, it follows that with a change in the parameters of the current strength I (A) and the pulse switching time $T_{on}\;$(μs), the value of the roughness purity Ra (μm) changes according to the quadratic dependence. It can also be seen that the change in the current I (A)has a much greater effect on the roughness index Ra (μm) than the change in the parameter $T_{on}\;$(μs). At the same time, the minimum value of Ra = 0.11 μm was achieved at I = 1 A and $T_{on}$ = 200 μs, and the maximum value Ra = 16.98 μmwas achieved at I = 7 A and $T_{on}$ = 90 μs.

An analysis of the data showed that the value of the current strength and, to a lesser extent, the duration of the action of the pulses have the greatest influence on the roughness of the treated surface. The voltage value has practically no effect on the roughness of the treated surface. The resulting model makes it possible to predict the value of the roughness of the surface of the 40Cr steel to be treated by EDM using an ET made of a composite copper–colloidal graphite material with a graphite content of 20%, depending on the processing modes.

### 3.2. Comparison of Experimental Results and Theoretical Model

Calculations of the dependence of the formed roughness on chromium–nickel steel were carried out according to the theoretical and experimental models.

Measurements of the holes of the working surfaces of the workpieces were carried out using an Olympus GX 51electron microscope. As a result, the average values of the diameters of the wells in μm were obtained and are presented in Table 6.

The mathematical model of the surface of the workpiece is a hole obtained as a result of the action of a single impulse, depending on the specified modes.

The table shows that the smallest values of the average diameters of the holes were obtained when carrying out experiments 1, 3, 5, 7, and 9, at which the values of the current strength are minimal (I = 2A). Based on the graph, one can see that when the current strength I changes in the range from 1A to 9A, the average values of the holes will change by 75%. With a change in the voltage values U, the diameter of the wells changes by an average of 8%. When changing the values of the duration of the pulse $T_{on}$, the diameter of the wells changes on average by 7%.

**Figure 10:**
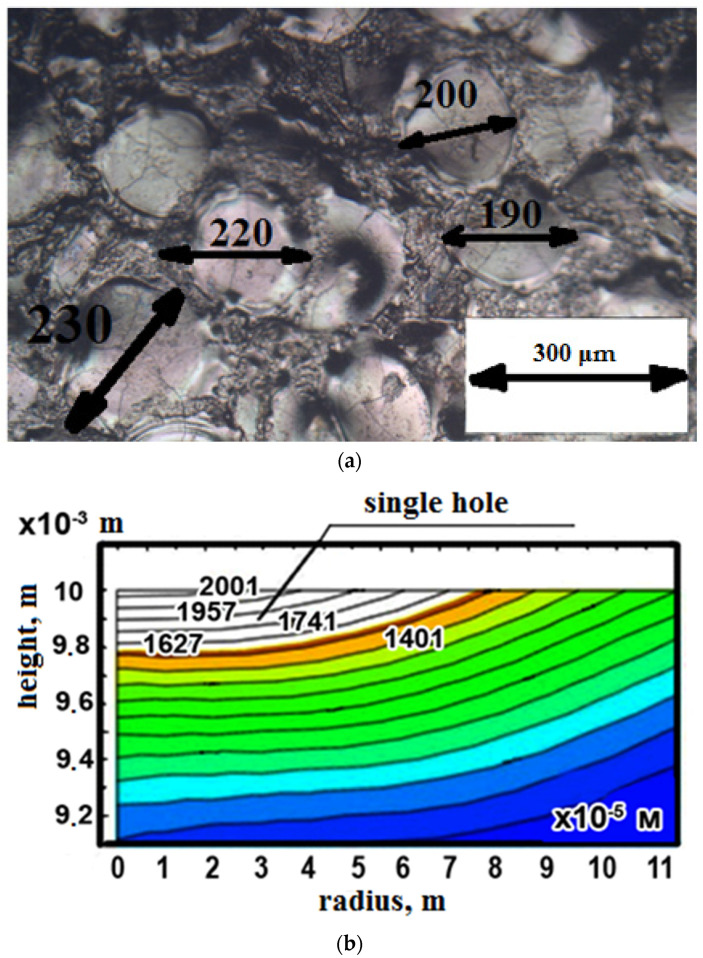
Comparative analysis of the sizes of single holes in mode No. 8 (max) for time $T_{on}=150$ μs: (**a**) the results of the experimental study (×200); and (**b**) the results of mathematical modeling.

Figure 10 shows photographs of working surfaces. Figure 10a is the surface obtained as a result of the experiment, and Figure 10b is the surface obtained as a result of modeling.

**Figure 11:**
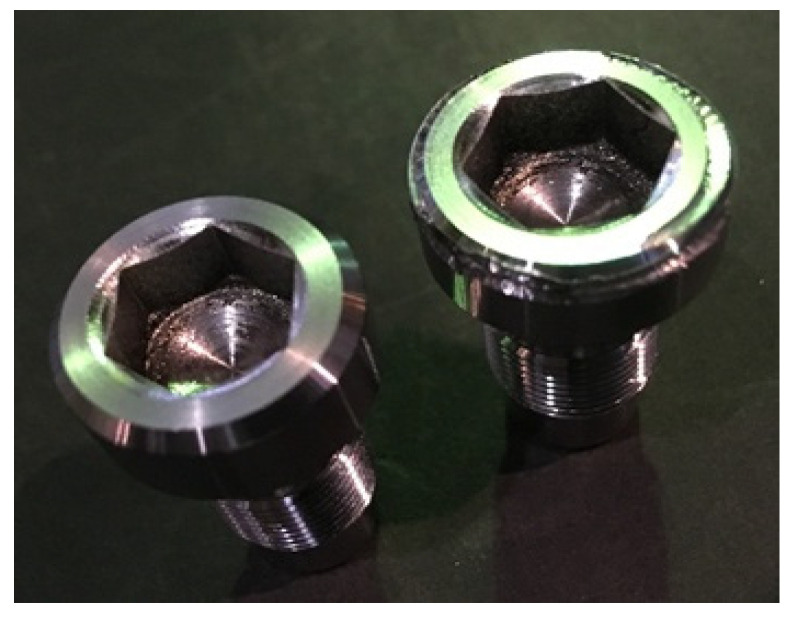
Detail of the “screw” part.

To compare the experimental and mathematical data, based on the matrix of orthogonal compositional planning of the experiment, the maximum (max) mode (experiment No. 8) was selected. Analyzing Figure 11, it can be seen that as a result of experiment No. 8 (the max mode), the deviation in the experimental value of the hole diameter from the value of the mathematical model is 11%. Using the obtained technique for modeling the size of electro-erosion holes, it is possible, with the declared accuracy, depending on the power of the processing parameters, to predict the resulting roughness when changing the processing mode and choosing another steel material with increased high-temperature wear resistance.

Using the results of the research carried out, industrial tests of the composite material ET were carried out. The tests were carried out on the “screw” part (Figure 11). The part material consisted of 40Cr alloyed structural steel heat-strengthened to a hardness of 45.5–50 HRC.

The “screw” part refers to a non-standard piece of hardware and is a responsible fastener that ensures the connection of parts in the bolt group of an artillery gun. The gate group is one of the most loaded units of the product; therefore, the parts included in this group must have an increased margin of safety since they operate under high loads and temperatures. The “screw” part is made of rolled stock. The enlarged production route is presented in Table 7.

The technological process of manufacturing the “screw” part includes the operation of electrical discharge machining, in which a hexagon with dimensions of 10 × 11.45 mm is burned on a 4E723 machine. An ET made from M1 GOST 589-2001 copper is used as a tool. Processing is carried out in technologically advanced modes, and the machine processing time is 14 min, 28 s. For testing, a composite material ET was made in which the content of graphite was 20%.

Based on the requirement in the design documentation for the “screw” part of a surface roughness Ra = 6.3 microns, using the obtained empirical model, the optimal parameters of the processing modes were determined and are shown in Table 8.

Further, copy-piercing electro-discharge machining of the hexagon in the “screw” ET part made of the specified composite material was performed, and the machine processing time was recorded.

After processing, a measurement of the geometric dimensions and the roughness of the resulting hexagon was made.

**Figure 12:**
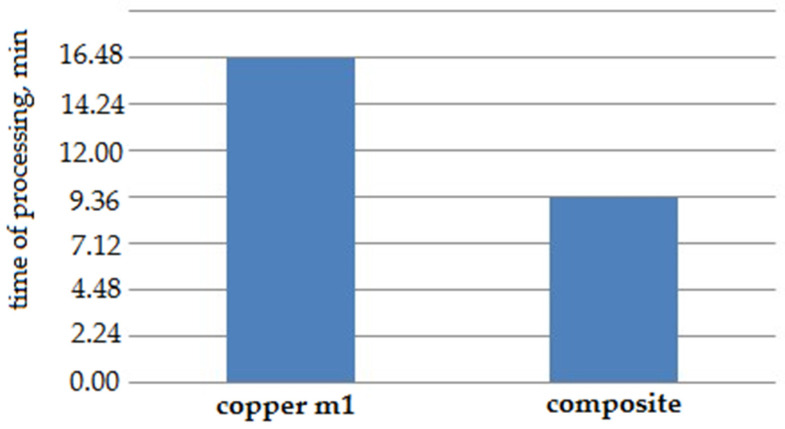
Machine processing time for the “screw” part.

As a result of the tests, it was found that the machine time for processing a hexagon ET from the composite material was 9 min 29 s, which is 35% less than the machine time when processing an ET from M1 copper (Figure 12). Measurements of the geometric parameters of the obtained hexagon show that all dimensions meet the requirements specified in the design documentation. The resulting roughness purity was Ra = 5.5 µm.

In general, the surface of the “screw” part obtained with the ETmade from the copper–colloidal graphite composite material practically did not differ qualitatively from the surface obtained with the ET made from M1 copper.

## 4. Conclusions

A theoretical model was developed that makes it possible to evaluate the effect of EDM modes on the roughness parameter of the EDM of parts using a 40Cr alloy steel ET made from a composite material, such as a pseudo-alloy system of copper–colloidal graphite with a graphite content of 20%.

An empirical model was developed that makes it possible to predict the effect of EDM modes on the roughness of the treated surface of parts using a 40Cr alloy steel ET made from a composite material, such as a pseudo-alloy system of copper–colloidal graphite with a graphite content of 20%.

Using the results of the research carried out and the obtained empirical models, an industrial test of an ET made from a copper–colloidal graphite composite material with a graphite content of 20% was carried out. A 35% reduction in the machine time was achieved when processing the “screw” part with the required surface quality indicators. Roughing was carried out at a current I = 9A, a voltage U = 100 V, and a pulse duration $T_{on}$ = 200 μs. Finishing was carried out at a current I = 2A, a voltage U = 85 V, and a pulse duration $T_{on}$ = 160 μs.

## Figures and Tables

**Table 1:** Thermal parameters of 40Cr structural steel.

P/p No.	Parameter	Designation	The quantity	Dimension
1	Heat capacity	Wed	460	j/(kg * °C)
2	Density	ρ	7820	kg/m
3	Coef.of thermal conductivity	k	44	wt/(m * °C)
4	Melting temperature	TPL	1550	°C
5	Boiling temperature	Tkip	2900	°C

**Table 2:** Process variables and their coded and actual levels for the CCD design matrix.

Parameters Coded Values	Actual Values of Coded Variables				
	Min Value (X1) (−1)	Max Value (X2) (+1)	Med Value(X0) (0)	Axial Value (-α) (−1.215)	Axial Value (α) (+1.215)
Current, I (A)	2	8	5	1	9
Pulseturn-on time, $T_{on}$ (µs)	40	150	100	30	200
Voltage, U (V)	50	100	75	45	105

**Table 3:** Initial parameters of the planning matrix.

No.	X1 (I, A)	$X2\;(T_{\mathrm{on}},\;\mu s)$	X3 (U, V)
1	2	40	50
2	8	40	50
3	2	150	50
4	8	150	50
5	2	40	100
6	8	40	100
7	2	150	100
8	8	150	100
9	1	100	75
10	9	100	75
11	5	30	75
12	5	200	75
13	5	100	45
14	5	100	105
15	5	100	75

**Table 4:** Experimental results, Ra (μm).

No.	Results			$\mathrm{Average},\;{\bar{y}}_{j}$	$\mathrm{Sample}\;\mathrm{Variance},\;S_{j}^{2}$	$\mathrm{Error},\;S_{j}$
	y1	y2	y3			
1	0.80	2.91	5.05	2.9200	4.515700	2.12502
2	10.46	13.91	7.04	10.4700	11.799300	3.43501
3	1.46	2.95	4.47	2.9600	2.265100	1.50502
4	13.48	6.96	10.19	10.2100	10.627900	3.26005
5	1.34	2.88	4.42	2.8800	2.371600	1.54000
6	13.96	7.14	10.52	10.5400	11.628400	3.41004
7	4.41	2.99	1.57	2.9900	2.016400	1.42000
8	14.88	11.01	7.14	11.0100	14.976900	3.87000
9	2.92	1.43	4.41	2.9200	2.220100	1.49000
10	12.43	8.38	16.50	12.4367	16.483633	4.06000
11	7.24	13.96	10.60	10.6000	11.289600	3.36000
12	7.35	10.65	13.95	10.6500	10.890000	3.30000
13	11.60	15.33	7.87	11.6000	13.912900	3.73000
14	15.57	7.67	11.62	11.6200	15.602500	3.95000
15	7.69	11.31	14.93	11.3100	13.104400	3.62000

**Table 5:** Coefficients of the polynomial.

Regression Coefficient	Coefficient	Dispersion of Reproducibility	Student’s Criterion
b0	4.15040453	2.0373	4.0943
b1	0.87471712	0.9353	4.1044
b2	0.87471712	0.9353	0.0411
b3	0.87471712	0.9353	0.0863
b12	1.19753694	1.0943	0.0069
b13	1.19753694	1.0943	0.1005
b23	1.19753694	1.0943	0.0914
b11	2.19660859	1.4821	2.2744
b22	2.19660859	1.4821	0.9285
b33	2.19660859	1.4821	0.4786

**Table 6:** Average values of the diameters of the holes, depending on the processing modes.

Experiment Number	EDM Modes			Average Value of Hole Diameters (μm)
	I(A)	$T_{on}(\mathrm{ms})$	U(V)	
1	2	40	50	62.45
2	8	40	50	195.784
3	2	150	50	58.556
4	8	150	50	190.970
5	2	40	100	48.230
6	8	40	100	205.396
7	2	150	100	58.428
8	8	150	100	170.478
9	1	100	75	50.935
10	9	100	75	245.998
11	5	300	75	226.247
12	5	200	75	223.576
13	5	100	45	219.378
14	5	100	105	237.118
15	5	100	75	216.305

**Table 7:** Enlarged technological route for the manufacture of the “screw” part.

Op. No.	Operation Name	Equipment	Brief Description of the Operation
005	Incoming control		Control of the workpiece parameters
010	Detachable	MEBA 260AP	Cutting of billets from rolled products
015	Heat treatment		Heat treatment for hardness (45.5–50 HRC)
020	Control		Hardness control
025	CNC turning	HardingeGS150	External geometry machining and threading
030	CNC turning	Hardinge GS150	Outside geometry machining, face trimming, and hex hole drilling
035	Electrical discharge	4E723	Copy-piercing EDM machining of a 10 × 11.45 mm hexagon.
040	Locksmith		Locksmith stripping
045	Control		Geometry control in accordance with design documentation

**Table 8:** Modes of processing the ET made from a composite material.

Processing Mode	I (A)	$T_{on}\;(\mathrm{ms})$	U (V)
Rough	9	200	80
Finish	3	200	80

## Data Availability

Not applicable.
